# Bis(2-meth­oxy­anilinium) hexa­bromido­stannate(IV) dihydrate

**DOI:** 10.1107/S1600536813031681

**Published:** 2013-11-23

**Authors:** Hassen Chouaib, Sahel Karoui, Slaheddine Kamoun, Francois Michaud

**Affiliations:** a Laboratoire de Genie des Materiaux et Environnement, Ecole Nationale d’Ingenieurs de Sfax, BP 1173, Sfax, Tunisia; b Service commun d’analyse par diffraction des rayons X, Universite de Brest, 6, avenue Victor Le Gorgeu, CS 93837, F-29238 Brest cedex 3, France

## Abstract

The asymmetric unit of the title compound, (C_7_H_10_NO)_2_[SnBr_6_]·2H_2_O, contains one cation, one half-dianion and one lattice water mol­ecule. The [SnBr_6_]^2−^ dianion, located on an inversion center, exhibits a highly distorted octa­hedral coordination environment, with Sn—Br bond lengths ranging from 2.2426 (9) to 3.0886 (13) Å. In the crystal, O—H⋯Br, N—H⋯Br, N—H⋯O and C—H⋯Br hydrogen bonds consolidate the packing, which can be described as consisting of alternating anionic (containing dianions and lattice water mol­ecules) and cationic layers parallel to *ab* plane.

## Related literature

For general background to hybrid organic–inorganic compounds, see: Kagan *et al.* (1999 ▶); Raptopoulou *et al.* (2002 ▶). For related structures, see: Tudela & Khan (1991 ▶); Chouaib *et al.* (2013 ▶); Benali-Cherif *et al.* (2007 ▶); Karoui *et al.* (2013 ▶); Guelmami *et al.* (2007 ▶); Souissi *et al.* (2011 ▶); Smith *et al.* (2006 ▶).
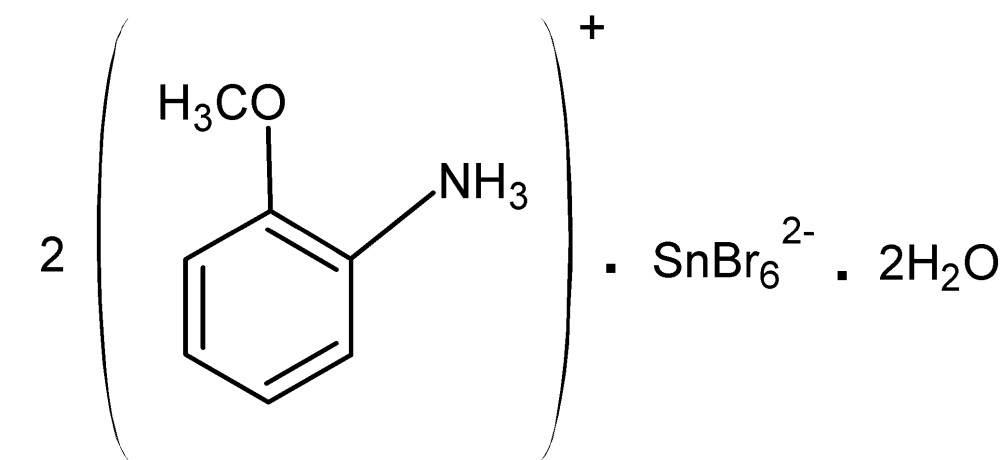


## Experimental

### 

#### Crystal data

(C_7_H_10_NO)_2_[SnBr_6_]·2H_2_O
*M* *_r_* = 882.50
Monoclinic, 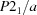

*a* = 10.8728 (7) Å
*b* = 13.4403 (10) Å
*c* = 9.0695 (6) Å
β = 103.680 (5)°
*V* = 1287.76 (15) Å^3^
*Z* = 2
Mo *K*α radiation
μ = 10.32 mm^−1^
*T* = 293 K
0.10 × 0.10 × 0.10 mm

#### Data collection

Agilent Xcalibur (Sapphire2) diffractometer
Absorption correction: multi-scan (*CrysAlis RED*; Agilent, 2012 ▶) *T*_min_ = 0.356, *T*_max_ = 0.371
7762 measured reflections
2188 independent reflections
1854 reflections with *I* > 2σ(*I*)
*R*_int_ = 0.052

#### Refinement

*R*[*F* ^2^ > 2σ(*F* ^2^)] = 0.061
*wR*(*F* ^2^) = 0.156
*S* = 1.07
2188 reflections
133 parameters
9 restraints
H atoms treated by a mixture of independent and constrained refinement
Δρ_max_ = 1.51 e Å^−3^
Δρ_min_ = −1.44 e Å^−3^

### 

Data collection: *CrysAlis PRO* (Agilent, 2012 ▶); cell refinement: *CrysAlis PRO*; data reduction: *CrysAlis RED* (Agilent, 2012 ▶); program(s) used to solve structure: *SHELXS97* (Sheldrick, 2008 ▶); program(s) used to refine structure: *SHELXL97* (Sheldrick, 2008 ▶); molecular graphics: *DIAMOND* (Brandenburg *et al.*, 1999 ▶) and *Mercury* (Macrae *et al.*, 2008 ▶); software used to prepare material for publication: *WinGX* (Farrugia, 2012 ▶).

## Supplementary Material

Crystal structure: contains datablock(s) I, New_Global_Publ_Block. DOI: 10.1107/S1600536813031681/cv5437sup1.cif

Structure factors: contains datablock(s) I. DOI: 10.1107/S1600536813031681/cv5437Isup2.hkl

Supplementary material file. DOI: 10.1107/S1600536813031681/cv5437Isup3.cdx

Additional supplementary materials:  crystallographic information; 3D view; checkCIF report

## Figures and Tables

**Table 1:** Hydrogen-bond geometry (Å, °)

*D*—H⋯*A*	*D*—H	H⋯*A*	*D*⋯*A*	*D*—H⋯*A*
N1—H1*B*⋯Br2^i^	0.89	2.82	3.557 (9)	141
N1—H1*C*⋯O*W* ^ii^	0.89	2.63	3.151 (15)	119
O*W*—H1*W*⋯Br2^i^	0.88 (9)	2.64 (9)	3.457 (9)	154 (11)
O*W*—H2*W*⋯Br1^iii^	0.88 (9)	2.42 (9)	3.296 (8)	171 (16)
C7—H7*A*⋯Br1^iii^	0.96	2.71	3.459 (15)	135
N1—H1*A*⋯Br1^ii^	0.89	2.75	3.154 (9)	109
C5—H5⋯Br2^iv^	0.93	2.52	3.289 (12)	140

Symmetry codes: (i) 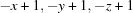; (ii) 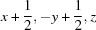; (iii) 
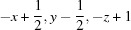
; (iv) 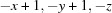.

**Fig. 1.**
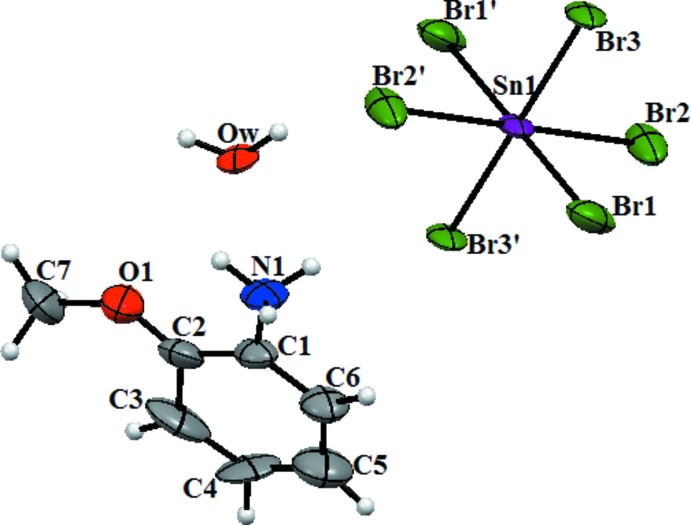
A view of the asymmetric unit of (I) showing the atomic numbering and 50% probability displacement ellipsoids [symmetry code: (i) -x + 1, -y + 1, -z + 1].

**Fig. 2.**
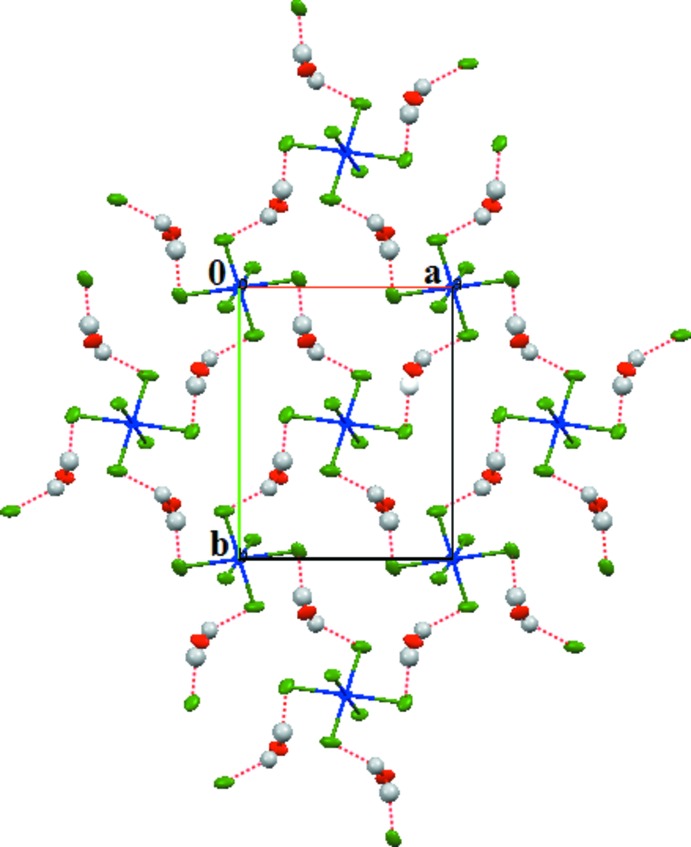
An anionic layer in (I) viewed along the c axis. Intermolecular hydrogen bonds are shown as red dashed lines.

**Fig. 3.**
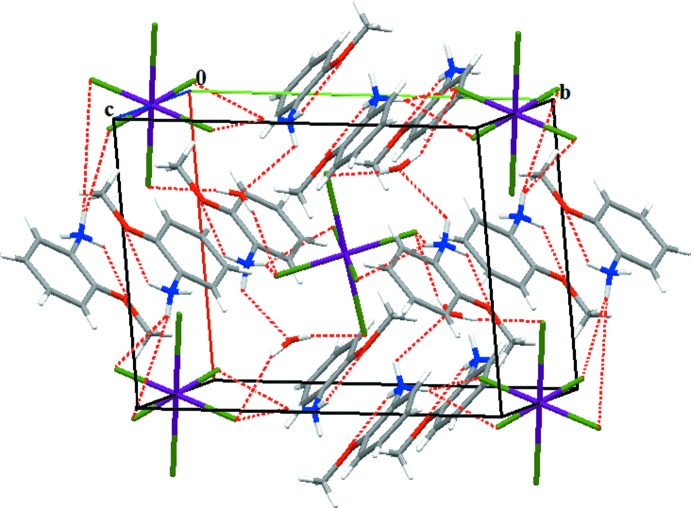
A portion of the crystal packing showing the hydrogen bonds as red dashed lines.

## supplementary crystallographic information

### 1. Comment

Hybrid organic-inorganic compounds are of great interest owing to their ionic, electrical, magnetic and optical properties (Kagan *et al.*, 1999; Raptopoulou *et al.*, 2002). As a continuation of our structural study of such hydrid compounds containing 2-methoxyanilinium cation (Karoui *et al.*, 2013), we report herein the crystal structure of the title compound (I).

The asymmetric unit of (I) contains one 2-methoxyanilinium cation, one-half dianion and one crystalline water molecule (Fig. 1). The [SnBr_6_]^2-^ dianion located on an inversion center exhibits a highly distorted octahedral coordination environment with Sn—Br bond lengths ranging from 2.2426 (9) Å to 3.0886 (13) Å. The Br—Sn—Br angles for the Br atoms in *cis* positions with respect to each other fall in the range of 83.74 (4) to 96.26 (4)°. The [SnBr_6_]^2-^ dianions interact with water molecules *via* O—H···Br hydrogen bonds (Table 1) thus forming anionic layers parallel to the *ab* plane (Fig. 2). Bond lengths and angles within the cations and dianions are as expected and comparable with those observed in the related compounds (Benali-Cherif *et al.*, 2007; Karoui *et al.*, 2013; Guelmami *et al.*, 2007; Souissi *et al.*, 2011; Smith *et al.*, 2006; Tudela & Khan, 1991; Chouaib *et al.*, 2013). The phenyl ring is practically planar with the greatest deviation from the six-atoms least squares plane being 0.0156 Å. The torsion angle O1—C1—C2—N1 is 2 (2)° indicating that the N1—C2 and C1—O1 groups deviate from the phenyl ring plane. The methoxy group of the organic cation makes an angle of 0(2)° with the plane of the phenyl ring and is in short intramolecular contact with O1 (N···O =2.824 (14) Å). The benzene ring is regular with C—C—C angles in agreement with the expected sp2 hybridation.

In the crystal, intermolecular O—H···Br, N—H···Br, N—H···O and C—H···Br hydrogen bonds (Table 1) consolidate the packing (Fig. 3), which can be described as consisting of alternating anionic (containing dianions and crystalline water molecules) and cationic layers parallel to *ab* plane.

### 2. Experimental

(C_7_H_10_NO)_2_SnBr_6_·2H_2_O was prepared by refluxing during 5 h a solution of metallic tin (3 g, 25 mmol) in 40 ml an aqueous solution of hydrobromic acid, HBr 47%. To this solution, 9.5 ml (75 mmol) of a solution of 2-methoxyanilin was added at reflux temperature. After a slow solvent evaporation yellow crystals suitable for X-ray analysis were obtained. They were washed with diethyl ether and dried over P_2_O_5_.

### 3. Refinement

All H atoms were geometrically positioned and treated as riding on their parent atoms, with C—H = 0.93 Å for the phenyl, 0.96 Å for the methyl and N—H= 0.89 Å with *U*_iso_(H)= 1.2 *U*_eq_(*C*-phenyl, N) or 1.5 *U*_eq_(*C*-methyl). The water H atoms were located in a difference Fourier map and refined using *DFIX* restraints (O—H 0.88 (7) Å). and a riding model, with *U*_iso_(H) = 1.5 *U*_eq_(O).

### Crystal data

**Table:**

(C_7_H_10_NO)_2_[SnBr_6_]·2H_2_O	*F*(000) = 828
*M**_r_* = 882.50	*D*_x_ = 2.276 Mg m^−^^3^*D*_m_ = 2.276 Mg m^−^^3^*D*_m_ measured by not measured
Monoclinic, *P*2_1_/*a*	Mo *K*α radiation, λ = 0.71073 Å
Hall symbol: -P 2yab	Cell parameters from 5970 reflections
*a* = 10.8728 (7) Å	θ = 3.6–24.7°
*b* = 13.4403 (10) Å	µ = 10.32 mm^−^^1^
*c* = 9.0695 (6) Å	*T* = 293 K
β = 103.680 (5)°	Prism, yellow
*V* = 1287.76 (15) Å^3^	0.10 × 0.10 × 0.10 mm
*Z* = 2	

### Data collection

**Table:**

Agilent Xcalibur (Sapphire2) diffractometer	1854 reflections with *I* > 2σ(*I*)
Radiation source: fine-focus sealed tube	*R*_int_ = 0.052
Graphite monochromator	θ_max_ = 24.7°, θ_min_ = 3.6°
ω scans	*h* = −12→12
Absorption correction: multi-scan (*CrysAlis RED*; Agilent, 2012)	*k* = −11→15
*T*_min_ = 0.356, *T*_max_ = 0.371	*l* = −10→10
7762 measured reflections	2 standard reflections every 120 min
2188 independent reflections	

### Refinement

**Table:**

Refinement on *F*^2^	Secondary atom site location: difference Fourier map
Least-squares matrix: full	Hydrogen site location: inferred from neighbouring sites
*R*[*F*^2^ > 2σ(*F*^2^)] = 0.061	H atoms treated by a mixture of independent and constrained refinement
*wR*(*F*^2^) = 0.156	*w* = 1/[σ^2^(*F*_o_^2^) + (0.0686*P*)^2^ + 18.8968*P*] where *P* = (*F*_o_^2^ + 2*F*_c_^2^)/3
*S* = 1.07	(Δ/σ)_max_ < 0.001
2188 reflections	Δρ_max_ = 1.51 e Å^−^^3^
133 parameters	Δρ_min_ = −1.44 e Å^−^^3^
9 restraints	Extinction correction: *SHELXL97* (Sheldrick, 2008), Fc^*^=kFc[1+0.001xFc^2^λ^3^/sin(2θ)]^-1/4^
Primary atom site location: structure-invariant direct methods	Extinction coefficient: 0.0039 (8)

### Special details

**Table:**

Geometry. All e.s.d.'s (except the e.s.d. in the dihedral angle between two l.s. planes) are estimated using the full covariance matrix. The cell e.s.d.'s are taken into account individually in the estimation of e.s.d.'s in distances, angles and torsion angles; correlations between e.s.d.'s in cell parameters are only used when they are defined by crystal symmetry. An approximate (isotropic) treatment of cell e.s.d.'s is used for estimating e.s.d.'s involving l.s. planes.
Refinement. Refinement of *F*^2^ against ALL reflections. The weighted *R*-factor *wR* and goodness of fit *S* are based on *F*^2^, conventional *R*-factors *R* are based on *F*, with *F* set to zero for negative *F*^2^. The threshold expression of *F*^2^ > σ(*F*^2^) is used only for calculating *R*-factors(gt) *etc*. and is not relevant to the choice of reflections for refinement. *R*-factors based on *F*^2^ are statistically about twice as large as those based on *F*, and *R*- factors based on ALL data will be even larger.

### Fractional atomic coordinates and isotropic or equivalent isotropic displacement parameters (Å^2^)

**Table:**

	*x*	*y*	*z*	*U*_iso_*/*U*_eq_	
Sn1	0.5000	0.5000	0.5000	0.0316 (4)	
Br2	0.42826 (15)	0.67591 (9)	0.41106 (12)	0.0609 (5)	
Br3	0.56362 (12)	0.57192 (8)	0.72849 (10)	0.0444 (4)	
Br1	0.22150 (13)	0.46965 (11)	0.51438 (14)	0.0582 (5)	
OW	0.3159 (10)	0.1977 (7)	0.3700 (8)	0.056 (2)	
N1	0.5673 (10)	0.1749 (8)	0.2638 (9)	0.047 (2)	
H1A	0.5324	0.1285	0.3109	0.071*	
H1B	0.5596	0.2341	0.3048	0.071*	
H1C	0.6489	0.1611	0.2738	0.071*	
O1	0.3632 (11)	0.0449 (7)	0.1485 (10)	0.068 (3)	
C1	0.5131 (11)	0.1763 (8)	0.1279 (11)	0.043 (3)	
C2	0.4114 (12)	0.1068 (9)	0.0787 (10)	0.050 (3)	
C6	0.5699 (11)	0.2478 (9)	0.0605 (10)	0.052 (3)	
H6	0.6399	0.2847	0.1101	0.063*	
C4	0.4156 (15)	0.1880 (12)	−0.1337 (13)	0.072 (4)	
H4	0.3809	0.1929	−0.2375	0.086*	
C7	0.2509 (19)	−0.0282 (12)	0.0978 (18)	0.081 (5)	
H7A	0.2398	−0.0658	0.1838	0.122*	
H7B	0.2682	−0.0728	0.0225	0.122*	
H7C	0.1751	0.0086	0.0557	0.122*	
C3	0.3580 (13)	0.1144 (11)	−0.0702 (11)	0.077 (5)	
H3	0.2914	0.0753	−0.1227	0.092*	
C5	0.5122 (16)	0.2580 (13)	−0.0858 (11)	0.081 (5)	
H5	0.5338	0.3061	−0.1489	0.097*	
H1W	0.360 (10)	0.238 (7)	0.439 (10)	0.08 (5)*	
H2W	0.297 (16)	0.139 (6)	0.402 (14)	0.10 (6)*	

### Atomic displacement parameters (Å^2^)

**Table:**

	*U*^11^	*U*^22^	*U*^33^	*U*^12^	*U*^13^	*U*^23^
Sn1	0.0520 (7)	0.0323 (6)	0.0091 (5)	0.0004 (4)	0.0045 (4)	−0.0037 (3)
Br2	0.1150 (12)	0.0381 (7)	0.0191 (6)	0.0070 (6)	−0.0052 (6)	0.0030 (4)
Br3	0.0776 (9)	0.0429 (7)	0.0101 (5)	−0.0015 (5)	0.0054 (5)	−0.0050 (4)
Br1	0.0642 (8)	0.0749 (9)	0.0411 (7)	−0.0200 (7)	0.0237 (6)	−0.0303 (6)
OW	0.099 (7)	0.055 (5)	0.015 (3)	−0.019 (5)	0.015 (4)	0.005 (3)
N1	0.065 (6)	0.057 (6)	0.018 (4)	0.006 (5)	0.005 (4)	0.007 (4)
O1	0.106 (8)	0.061 (6)	0.038 (5)	−0.027 (5)	0.021 (5)	−0.010 (4)
C1	0.060 (7)	0.048 (6)	0.020 (5)	0.007 (5)	0.010 (5)	0.004 (4)
C2	0.066 (8)	0.059 (8)	0.031 (6)	−0.001 (6)	0.021 (5)	−0.016 (5)
C6	0.066 (8)	0.063 (8)	0.023 (5)	0.001 (6)	0.000 (5)	0.000 (5)
C4	0.088 (10)	0.113 (13)	0.016 (5)	0.000 (9)	0.015 (6)	0.020 (7)
C7	0.131 (15)	0.061 (9)	0.057 (9)	−0.034 (9)	0.035 (9)	−0.032 (7)
C3	0.068 (9)	0.113 (13)	0.041 (7)	0.001 (9)	−0.004 (6)	−0.048 (8)
C5	0.104 (12)	0.100 (12)	0.035 (7)	−0.003 (10)	0.010 (7)	−0.012 (8)

### Geometric parameters (Å, º)

**Table:**

Sn1—Br3	2.2426 (9)	O1—C7	1.550 (18)
Sn1—Br3^i^	2.2426 (9)	C1—C6	1.363 (9)
Sn1—Br2	2.5595 (11)	C1—C2	1.437 (16)
Sn1—Br2^i^	2.5595 (11)	C2—C3	1.341 (9)
Sn1—Br2^i^	2.5595 (11)	C6—C5	1.333 (9)
Sn1—Br1	3.0886 (13)	C6—H6	0.9300
Sn1—Br1^i^	3.0886 (13)	C4—C3	1.368 (9)
OW—H1W	0.88 (9)	C4—C5	1.40 (2)
OW—H2W	0.88 (9)	C4—H4	0.9300
N1—C1	1.234 (13)	C7—H7A	0.9600
N1—H1A	0.8900	C7—H7B	0.9600
N1—H1B	0.8900	C7—H7C	0.9600
N1—H1C	0.8900	C3—H3	0.9300
O1—C2	1.235 (14)	C5—H5	0.9300
			
Br3—Sn1—Br3^i^	180.0	H1B—N1—H1C	109.5
Br3—Sn1—Br2	84.12 (4)	C2—O1—C7	132.4 (11)
Br3^i^—Sn1—Br2	95.88 (4)	N1—C1—C6	107.4 (10)
Br3—Sn1—Br2^i^	95.88 (4)	N1—C1—C2	116.8 (9)
Br3^i^—Sn1—Br2^i^	84.12 (4)	C6—C1—C2	135.8 (9)
Br2—Sn1—Br2^i^	180.0	O1—C2—C3	115.7 (12)
Br3—Sn1—Br2^i^	95.88 (4)	O1—C2—C1	131.9 (9)
Br3^i^—Sn1—Br2^i^	84.12 (4)	C3—C2—C1	112.4 (10)
Br2—Sn1—Br2^i^	180.0	C5—C6—C1	111.2 (11)
Br2^i^—Sn1—Br2^i^	0.00 (5)	C5—C6—H6	124.4
Br3—Sn1—Br1	96.26 (4)	C1—C6—H6	124.4
Br3^i^—Sn1—Br1	83.74 (4)	C3—C4—C5	137.6 (11)
Br2—Sn1—Br1	84.64 (5)	C3—C4—H4	111.2
Br2^i^—Sn1—Br1	95.36 (5)	C5—C4—H4	111.2
Br2^i^—Sn1—Br1	95.36 (5)	O1—C7—H7A	109.5
Br3—Sn1—Br1^i^	83.74 (4)	O1—C7—H7B	109.5
Br3^i^—Sn1—Br1^i^	96.26 (4)	H7A—C7—H7B	109.5
Br2—Sn1—Br1^i^	95.36 (5)	O1—C7—H7C	109.5
Br2^i^—Sn1—Br1^i^	84.64 (5)	H7A—C7—H7C	109.5
Br2^i^—Sn1—Br1^i^	84.64 (5)	H7B—C7—H7C	109.5
Br1—Sn1—Br1^i^	180.00 (5)	C2—C3—C4	110.3 (10)
H1W—OW—H2W	116.9 (4)	C2—C3—H3	124.8
C1—N1—H1A	109.5	C4—C3—H3	124.8
C1—N1—H1B	109.5	C6—C5—C4	112.5 (12)
H1A—N1—H1B	109.5	C6—C5—H5	123.8
C1—N1—H1C	109.5	C4—C5—H5	123.8
H1A—N1—H1C	109.5		
			
C7—O1—C2—C3	0 (2)	C2—C1—C6—C5	4 (2)
C7—O1—C2—C1	177.8 (14)	O1—C2—C3—C4	177.3 (13)
N1—C1—C2—O1	2 (2)	C1—C2—C3—C4	−0.6 (17)
C6—C1—C2—O1	−178.2 (15)	C5—C4—C3—C2	−2 (3)
N1—C1—C2—C3	179.6 (12)	C1—C6—C5—C4	−4.5 (19)
C6—C1—C2—C3	−1 (2)	C3—C4—C5—C6	5 (3)
N1—C1—C6—C5	−176.6 (13)		

Symmetry code: (i) −*x*+1, −*y*+1, −*z*+1.

### Hydrogen-bond geometry (Å, º)

**Table:**

*D*—H···*A*	*D*—H	H···*A*	*D*···*A*	*D*—H···*A*
N1—H1*B*···Br2^i^	0.89	2.82	3.557 (9)	141
N1—H1*C*···O*W*^ii^	0.89	2.63	3.151 (15)	119
O*W*—H1*W*···Br2^i^	0.88 (9)	2.64 (9)	3.457 (9)	154 (11)
O*W*—H2*W*···Br1^iii^	0.88 (9)	2.42 (9)	3.296 (8)	171 (16)
C7—H7*A*···Br1^iii^	0.96	2.71	3.459 (15)	135
N1—H1*A*···Br1^ii^	0.89	2.75	3.154 (9)	109
C5—H5···Br2^iv^	0.93	2.52	3.289 (12)	140

Symmetry codes: (i) −*x*+1, −*y*+1, −*z*+1; (ii) *x*+1/2, −*y*+1/2, *z*; (iii) −*x*+1/2, *y*−1/2, −*z*+1; (iv) −*x*+1, −*y*+1, −*z*.
